# B cells do not have a major pathophysiologic role in acute ischemic stroke in mice

**DOI:** 10.1186/s12974-017-0890-x

**Published:** 2017-06-02

**Authors:** Michael K. Schuhmann, Friederike Langhauser, Peter Kraft, Christoph Kleinschnitz

**Affiliations:** 1 Department of Neurology, University Hospital Würzburg, Würzburg, Germany; 2 Department of Neurology, University Hospital Essen, 45147 Essen, Germany

**Keywords:** Ischemic stroke, Transient middle cerebral artery occlusion, B cells

## Abstract

**Background:**

Lymphocytes have been shown to play an important role in the pathophysiology of acute ischemic stroke, but the properties of B cells remain controversial. The aim of this study was to unravel the role of B cells during acute cerebral ischemia using pharmacologic B cell depletion, B cell transgenic mice, and adoptive B cell transfer experiments.

**Methods:**

Transient middle cerebral artery occlusion (60 min) was induced in wild-type mice treated with an anti-CD20 antibody 24 h before stroke onset, *JHD* ^*−/−*^ mice and *Rag1* ^*−/−*^ mice 24 h after adoptive B cell transfer. Stroke outcome was assessed at days 1 and 3. Infarct volumes were calculated from 2,3,5-triphenyltetrazolium chloride (TTC)-stained brain sections, and neurological scores were evaluated. The local inflammatory response was determined by real-time PCR and immunohistochemistry. Apoptosis was analyzed by TUNEL staining, and astrocyte activation was revealed using immunohistochemistry and Western blot.

**Results:**

Pharmacologic depletion of B cells did not influence infarct volumes and functional outcome at day 1 after stroke. Additionally, lack of circulating B cells in *JHD* ^*−/−*^ mice also failed to influence stroke outcome at days 1 and 3. Furthermore, reconstitution of *Rag1* ^*−/−*^ mice with B cells had no influence on infarct volumes.

**Conclusion:**

Targeting B cells in experimental stroke did not influence lesion volume and functional outcome during the acute phase. Our findings argue against a major pathophysiologic role of B cells during acute ischemic stroke.

**Electronic supplementary material:**

The online version of this article (doi:10.1186/s12974-017-0890-x) contains supplementary material, which is available to authorized users.

## Introduction

The inflammatory response after ischemic stroke (IS) is being increasingly investigated, and modulating inflammation might become an important treatment strategy. It became apparent that in acute stroke, lymphocytes transmigrate from the blood across the cerebral endothelium into the brain parenchyma [1, 2]. Moreover, there is increasing evidence that T cells are the key players in the pathophysiology of IS. In 2006, Yilmaz and co-workers first showed that *Rag1* ^*−/−*^ mice, i.e., animals lacking B and T cells, after adoptive transfer (AT) of T cells develop stroke volumes like wild-type (WT) animals, while *Rag1* ^*−/−*^ animals without AT are protected from IS [3].

Although the detrimental role of non-regulatory T cells on acute IS has been unequivocally proven, the impact of B cells is incompletely understood. Scientific reports showed discrepant results: some found a beneficial role of B cells [4–6] others found no impact on stroke volume and functional outcome [3, 7]. Doyle et al. reported a deleterious role of B cells on long-term cognitive function [8].

Our study’s aim was to further elucidate the pathogenic importance of B cells focusing on the acute phase of IS development using three experimental approaches (pharmacologic, transgenic mice, and AT experiments).

## Materials and methods

### Animals, sample size calculation

In this study, male C57BL/6, *JHD* ^*−/−*^ [9], *JHD* ^*+/+*^, or *Rag1* ^*−/−*^ [10] mice with an age of 12–16 weeks were used. All animal experiments were approved by local state authorities (Regierung von Unterfranken) and performed in accordance with the Animal Research: Reporting In Vivo Experiments (ARRIVE) guidelines (http://www.nc3rs.org.uk/ARRIVE). All mice were randomly assigned to the operators by an independent person not involved in data acquisition and analysis. We performed surgery and evaluation of all read-out parameters while blinded to the experimental groups. Assuming a reduction of infarct volume of 30% as functionally relevant and a standard deviation of 20% to the respective mean values, a group size of 8–10 was necessary to show this effect with a power of 0.8 and a probability of a type I error of <0.5 (calculated with GraphPad StatMate 2.00).

### Animal treatment

To deplete B cells, mice received 10 mg/kg anti-mouse CD20 (clone 5D2, Genentech) 1 day before tMCAO. Anti-ragweed (mouse IgG2a, Genentech) served as control [11]. For B and T cell transfer experiments into *Rag1* ^*−/−*^ mice, splenic B and T cells were isolated by negative selection (Miltenyi Biotech). Cells were injected intravenously (750,000 cells/mouse) 1 day before tMCAO [7].

### tMCAO

Focal cerebral ischemia was induced in C57BL/6, *JHD* ^*−/−*^ [9], *JHD* ^*+/+*^, or *Rag1* ^*−/−*^ [10] mice by 60-min transient middle cerebral artery occlusion (tMCAO) as described previously [12]. Edema-corrected infarct volumes were calculated from brain slices stained with 2,3,5­- triphenyltetrazolium chloride. Mice dying within 24 h after tMCAO or with subarachnoid hemorrhage or bleeding (as assessed macroscopically during brain sampling) were excluded from end-point analyses (Additional file 1: Table S1). The Bederson score and the grip test score were used to monitor neurologic function [13, 14].

### Protein extraction and Western blot analysis

Western blot analysis was performed according to standard procedures using a monoclonal antibody against glial fibrillary acidic protein (GFAP; ab7260; Abcam) and anti-­β-­actin (A5441; Sigma-Aldrich) [15].

### Real-time polymerase chain reaction

Tissue homogenization, RNA isolation, and real-time PCR were performed as described recently [16]. Relative gene expression levels of tumor necrosis factor-α (TNFα) (assay ID: Mm 00443258_m1, Applied Biosystems), interleukin (IL)-1β (assay ID: Mm 00434228_m1, Applied Biosystems), and IL-10 (assay ID: Mm 00439616_m1, Applied Biosystems) were analyzed with a fluorescent TaqMan technology. As an endogenous control Gapdh (TaqMan® Predeveloped Assay Reagent for gene expression, part number: 4352339E, Applied Biosystems) was used. PCR was performed using the StepOnePlus™ Real-Time PCR System (Applied Biosystem).

### Immunohistochemistry

Immunohistochemistry and histology of cryoembedded brain slices were performed as described elsewhere [12] using the following antibodies: anti-mouse Ly6B (MCA771GA, Serotec), anti-mouse CD11b (MCA711, Serotec), anti-mouse GFAP (ab7260, Abcam), and anti-mouse NeuN (MAB377, Millipore). For quantification of early apoptotic cell death, the In Situ Cell Death Detection Kit (12156792910, Roche) was used according to the user’s manual. Comparable brain sections were selected, and cell counting was performed from four to five subsequent slices (distance 100 μm) per animal. For CD11b and Ly6B, the total number of cells per ipsilesional hemisphere was counted. The number of dead neurons (TUNEL/NeuN double positive cells) was analyzed by counting three optical fields per cortex and two optical fields per basal ganglial region. The sections were analyzed under a microscope (Nikon Eclipse 50i) equipped with a charge-coupled device camera using 20-fold magnification.

### Statistical analysis

For statistical analysis, the GraphPad Prism 5.0 software package (GraphPad Software) was used. Results are given as mean ± standard error of the mean except for the Bederson score and the grip test, which are expressed as ordinal values. Data were tested for Gaussian distribution with the D’Agostino and Pearson omnibus normality test and then analyzed by unpaired, two-tailed Student’s *t* test. Scores addressing the functional outcome were compared using the Mann–Whitney *U* test. One-way ANOVA with post hoc Bonferroni correction was applied when comparing more than two groups. *P* < 0.05 was considered statistically significant.

## Results

**Fig. 1:**
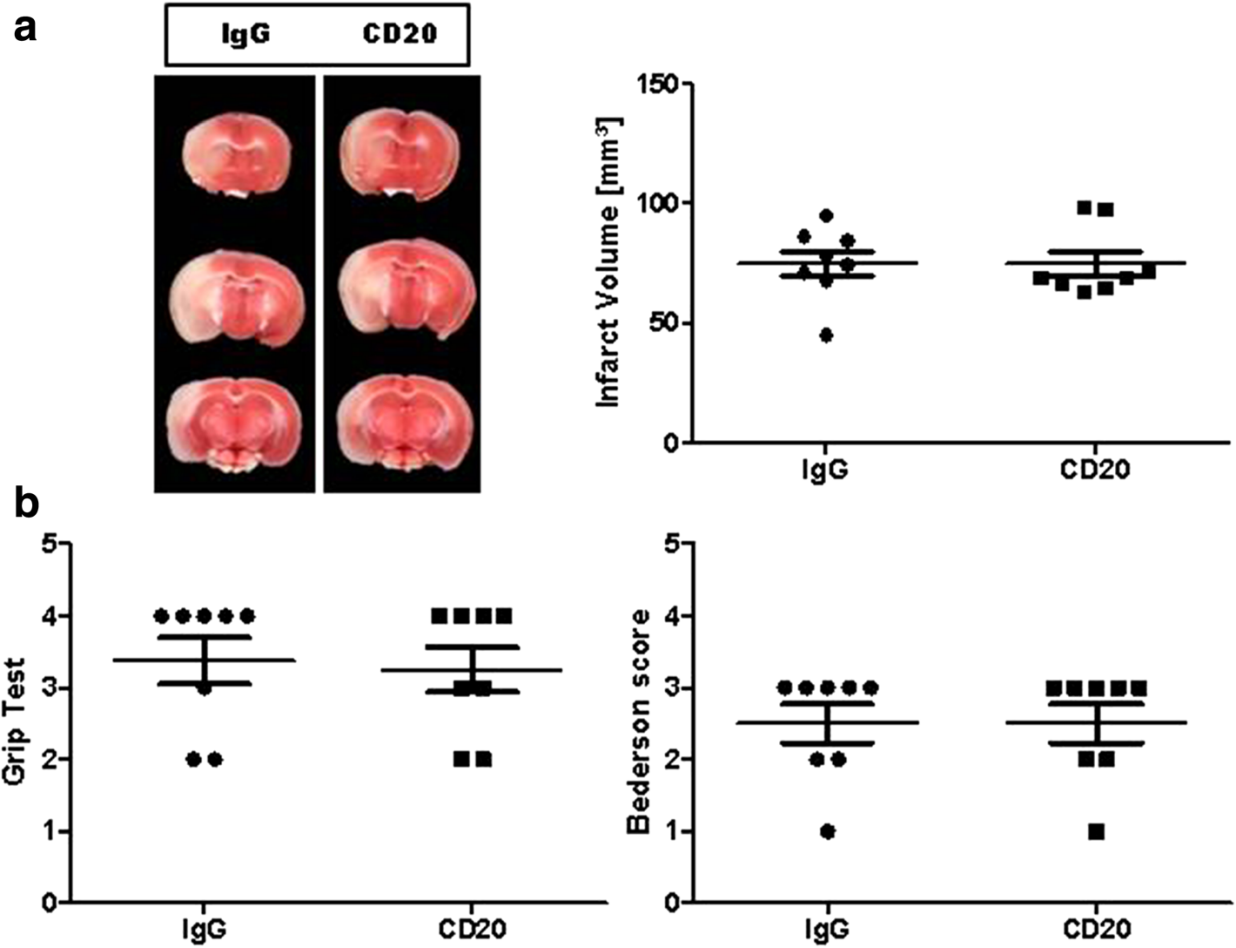
Pharmacologic prestroke B cell depletion does not alter stroke outcome in a transient middle cerebral artery occlusion (tMCAO) model. **a** *Left*, representative 2,3,5-triphenyltetrazolium chloride stains of three corresponding brain sections of an IgG-treated C57BL/6 mouse (IgG) and a C57BL/6 mouse at day 1 after stroke, treated with a specific antibody against CD20 1 day before 60-min tMCAO. **a** *Right*, infarct volumes are similar (*n* = 8/group) between the two treatment groups (unpaired, two-tailed Student’s *t* test). **b** B cell depletion does not improve functional outcome on day 1 after tMCAO (*n* = 8/group) as assessed by the grip test (*left*) and Bederson score (*right*), Mann–Whitney test

First, we confirmed that anti-CD20 treatment reduces circulating B cell numbers in mice, as previously described (Additional file 1: Figure S1) [9]. Next, we assessed if B cell depletion before tMCAO influences stroke development in WT mice. Stroke volumes (IgG 74.9 ± 5.4 mm3; CD20 74.7 ± 5.1 mm3; *P* > 0.05) (Fig. 1a) as well as functional outcome, as assessed by the grip test (values are the median with 25th and 75th percentiles, respectively, in brackets (IgG 4.0 (2.25, 4.0); CD20 3.5 (2.25, 4.0); *P* > 0.05), and the Bederson score (IgG 3.0 (2.0, 3.0); CD20 3.0 (2.0, 3.0); *P* > 0.05) (Fig. 1b) on day 1 were comparable with WT animals that had received isotype control antibodies.

**Fig. 2:**
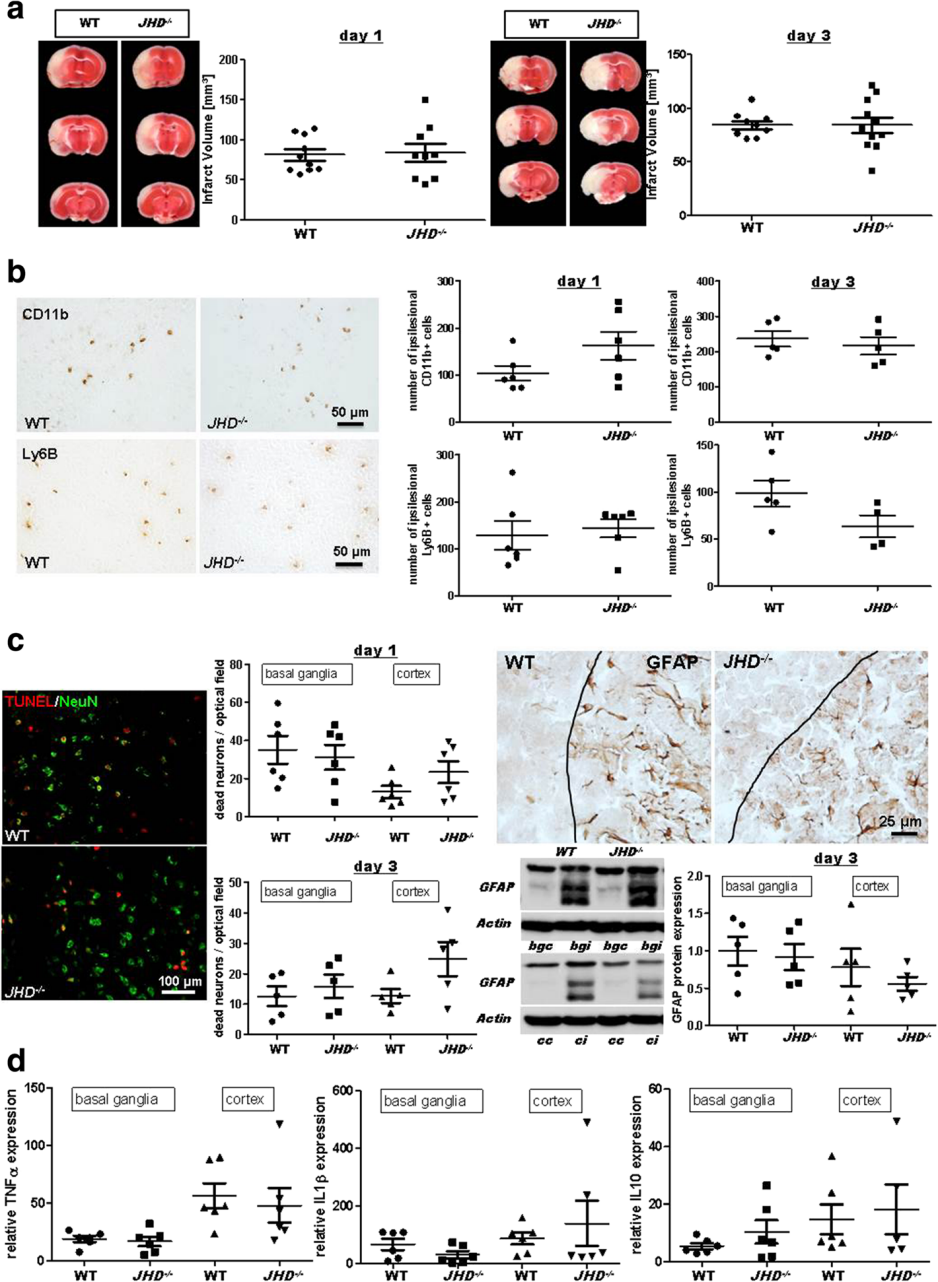
Lack of B cells does not impact stroke outcome in a transient middle cerebral artery occlusion (tMCAO) model. **a** Representative 2,3,5-triphenyltetrazolium chloride stains of three corresponding brain sections and infarct volumes of control (WT) and B cell-deficient (*JHD* ^*−/−*^) mice at days 1 (*left*) and 3 (*right*) after 60-min tMCAO (*n* = 9–11/group). Infarct volumes are similar between the two groups for both time points (unpaired, two-tailed Student’s *t* test). **b** *Left*, representative immunocytologic staining of CD11b^+^ and Ly6B^+^ cells within the ischemic brains of WT and *JHD* ^*−/−*^ mice. **b** *Right*, quantification revealed comparable numbers of CD11b^+^ monocytes and Ly6B^+^ neutrophils in the ipsilesional hemispheres of WT and *JHD* ^*−/−*^ mice at days 1 (*n* = 6/group) and 3 (*n* = 4–5/group) after tMCAO (unpaired, two-tailed Student’s *t* test). **c** *Left*, representative brain sections from a WT and a *JHD* ^*−/−*^ mouse stained for the neuronal marker NeuN and subjected to TUNEL assay to visualize apoptosis. Quantification of dead neurons per optical field in basal ganglial as well as cortical regions at days 1 (*n* = 6/group) and 3 (*n* = 5/group) was comparable between WT and *JHD* ^*−/−*^ mice (unpaired, two-tailed Student’s *t* test). **c** *Right*, representative immunocytologic staining of glial fibrillary acidic protein (*GFAP*) immunoreactivity in the penumbra (*black lines*) of the ischemic cortex of a WT and a *JHD* ^*−/−*^ mouse at day 3 after tMCAO (*top*). Representative anti-GFAP Western blot analysis (*cc* cortex contralesional, *ci* cortex ipsilesional, *bgc* basal ganglia contralesional, *bgi* basal ganglia ipsilesional) and densitometric quantification of ipsilesional GFAP protein expression in the basal ganglial as well as cortical regions at day 3 (*n* = 5/group) after tMCAO (*bottom*) was comparable between WT and *JHD* ^*−/−*^ mice (unpaired, two-tailed Student’s *t* test). **d** Relative gene expression of tumor necrosis factor α (TNFα), interleukin (IL)-1β, and IL-10 in the ischemic basal ganglia and cortex are similar at day 1 after tMCAO of WT and *JHD* ^*−/−*^ mice (*n* = 5–6/group) when normalized to sham operation (unpaired, two-tailed Student’s *t* test)

To exclude that the CD20 antibody influences outcome measures independent of its B cell-depleting effect, we additionally analyzed *JHD* ^*−/−*^ mice that lack B cells (Additional file 1: Figure S1) [10]. Again, there was no difference in infarct volumes when comparing *JHD* ^*−/−*^ with WT control mice at days 1 and 3 after stroke (day 1 WT, 81.2 ± 7.0 mm3; *JHD* ^*−/−*^, 84.2 ± 11.5 mm3; *P* > 0.05; day 3 WT, 84.4 ± 3.9 mm3; *JHD* ^*−/−*^, 84.1 ± 7.2 mm3; *P* > 0.05) (Fig. 2a). Importantly, cerebral blood flow was comparable in WT and *JHD* ^*−/−*^ mice before, during, and after tMCAO (Additional file 1: Figure S2). We next analyzed if the lack of B cells alters the composition of the cellular infiltrate within the ischemic brain. We found comparable ipsilesional numbers of CD11b^+^ monocytes and Ly6B^+^ neutrophils in control and *JHD* ^*−/−*^ mice at days 1 and 3 after stroke (Fig. 2b). We then analyzed neuronal apoptosis and GFAP expression levels (Fig. 2c). No impact of B cells on neuronal survival or astrocyte activation was observed. In line with these results, the expression levels of tumor necrosis factor α (TNFα), interleukin (IL)-1β, and IL-10 did not differ between WT and *JHD* ^*−/−*^ mice at day 1 (Fig. 2d).

**Fig. 3:**
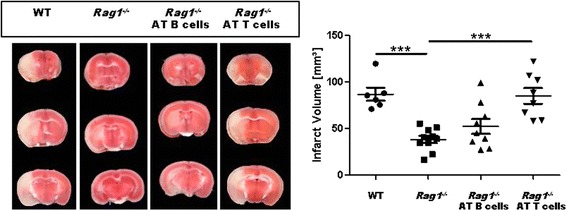
Prestroke B cell adoptive transfer (AT) into *Rag1* ^*−/−*^ mice does not worsen stroke outcome in a transient middle cerebral artery occlusion (tMCAO) model. *Left*, representative 2,3,5-­triphenyltetrazolium chloride stains of three corresponding brain sections of a C57BL/6 mouse (WT), a *Rag1* ^*−/−*^ mouse without adoptive cell transfer (*Rag1* ^*-/-*^) and with adoptively transferred B cells (*Rag1* ^*−/−>*^ AT B cells) or T cells (*Rag1* ^*−/−*^ AT B cells) on day 1 after 60-min tMCAO. *Right*, infarct volumes are reduced in *Rag1* ^*−/−*^ mice (*n* = 10) compared with those in WT mice (*n* = 6). Only AT of T cells (*n* = 8), but not B cells (*n* = 9), increased infarct volumes (one-way analysis of variance with post hoc Bonferroni adjustment for *P* values). ***P < 0.001

To further strengthen our finding that B cells do not play a significant role during the acute phase of ischemic lesion growth (as assessed by the use of pharmacologic depletion as well as genetic lack of B cells), we additionally performed B cell transfer experiments using *Rag1* ^*−/−*^ mice that lack B and T lymphocytes. While infarct volumes at day 1 after tMCAO were significantly smaller in *Rag1* ^*−/−*^ mice compared with those in WT mice (*P* < 0.001), AT of T cells (*P* < 0.001), but not of B cells (*P* > 0.05), significantly increased infarct volumes (Fig. 3).

## Discussion

In the present study, with the use of pharmacologic depletion of circulating B cells, *JHD* ^*−/−*^ mice, and AT experiments of B cells into *Rag1* ^*−/−*^ mice, we independently confirm that B cells play a minor role in acute IS. Pharmacologic and genetic depletion of B cells did not affect stroke size in all of the experimental approaches [3, 7, 8]. Also, transferring B cells into *Rag1* ^*−/−*^ mice did not neutralize the neuroprotective effect of immune deficiency although there was a slight increase of infarct volumes after AT of B cells what is consistent with previous studies [7]. Importantly, *Rag1* ^*−/−*^ mice supplemented with T cells were fully susceptible to ischemic brain damage, proving that T cells are detrimental in early stroke development.

Moreover, we did not find evidence that B cells contribute to the local inflammatory response because ipsilesional numbers of monocytes and neutrophils, cells that are well known to appear within the first 3 days after tMCAO [2], as well as expression levels of the pro-inflammatory cytokines TNFα and IL1β, were comparable in mice lacking B cells and corresponding WT controls. Consequently, neuronal apoptosis and astrocyte reactivity that seem to correlate with the degree of immune activity [2] were also indistinguishable in WT and B cell-deficient mice. In contrast to previous studies [3, 7, 8] and our results (all arguing for a negligible role of B cells during the acute phase of cerebral ischemia), it has been reported that B cells are cerebroprotective immunomodulators through anti-inflammatory effects [4, 6]. The reasons for the differences between these studies may partially be explained by the use of different experimental settings. While the Offner group [4, 6] used *μMT* ^*−/−*^ mice as a B cell-deficient mouse strain, we used the *JHD* ^*−/−*^ mice, and also, the number of B cells used for adoptive transfer are different (5 million vs 750.000). Moreover, the extent of brain damage (large infarctions vs moderate infarctions) might have differentially influenced the function of B cells and, therefore, stroke outcome [17].These discrepant findings clearly underline that research on the impact of B cells in stroke lies in its infancy, and it is still incompletely understood how the immune system is regulated and contributes to the pathophysiology of an ischemic insult. Besides the strengths of our investigation (using three independent approaches to investigate the influence of B cells in the acute phase of cerebral ischemia), additional studies are needed to finally evaluate the relevance of B cells in IS. B cell intervention should be studied in different stroke models as it is known that there could be differences in transient versus permanent stroke models without reperfusion. Furthermore, distinct time lines of B cell interventions should be investigated. In this study, AT of B cells was performed 1 day prior to experimental stroke and we cannot rule out that other time lines might cause other findings. For a better translational relevance, B cell intervention should also be addressed after the ischemic stimulus. In addition, the role of B cells in the later phase of post stroke recovery requires further investigations. To overcome the poor reproducibility of preclinical trials and poor translation to the clinic, multicenter preclinical randomized controlled trials (pRCTs) have been proposed as a suitable tool for “bridging the gap” between experimental research and clinical trials [18]. In such pRCTs, standardized protocols in collaboration with independent multinational research centers are used and such an approach appears best suitable to address such a challenging task.

The present study indicates that B cells play a minor role in infarct development during acute IS.

## Additional file

Additional file 1: Supplemental Results and Methods. (DOCX 1116 kb)

## Abbreviations

AT: Adoptive transfer
GFAP: Glial fibrillary acid protein
IL: Interleukin
IS: Ischemic stroke
pRCTs: Preclinical randomized controlled trials
tMCAO: Transient middle cerebral artery occlusion
TNF: Tumor necrosis factor
WT: Wild-type
