# Supplementary material for: B cells do not have a major pathophysiologic role in acute ischemic stroke in mice
Source: J Neuroinflammation. 2017 Jun 2;14:112. doi: 10.1186/s12974-017-0890-x (PMC5457733; doi:10.1186/s12974-017-0890-x)
Supplement: Additional file 1: — Supplemental Results and Methods. (DOCX 1116 kb) [file 12974_2017_890_MOESM1_ESM.docx]

**SUPPLEMENT**

**B Cells Do Not Have a Major Pathophysiologic Role in Acute Ischemic Stroke in Mice**

Michael K. Schuhmann^1^, Friederike Langhauser^1^, Peter Kraft^1,†^ and Christoph Kleinschnitz^1,2,†,*^

^1^Department of Neurology, University Hospital Würzburg, Würzburg, Germany

^2^Department of Neurology, University Hospital Essen, Essen, Germany

^†^Equal contribution.

*Correspondence: Christoph Kleinschnitz, MD, University Clinic Essen, Department of Neurology, Hufelandstraße 55, 45147 Essen, Germany. Tel.: +49-201-723-6302; Fax: +49-201-723-5655, E-mail: [christoph.kleinschnitz@uk-essen.de](mailto:christoph.kleinschnitz@uk-essen.de)

**
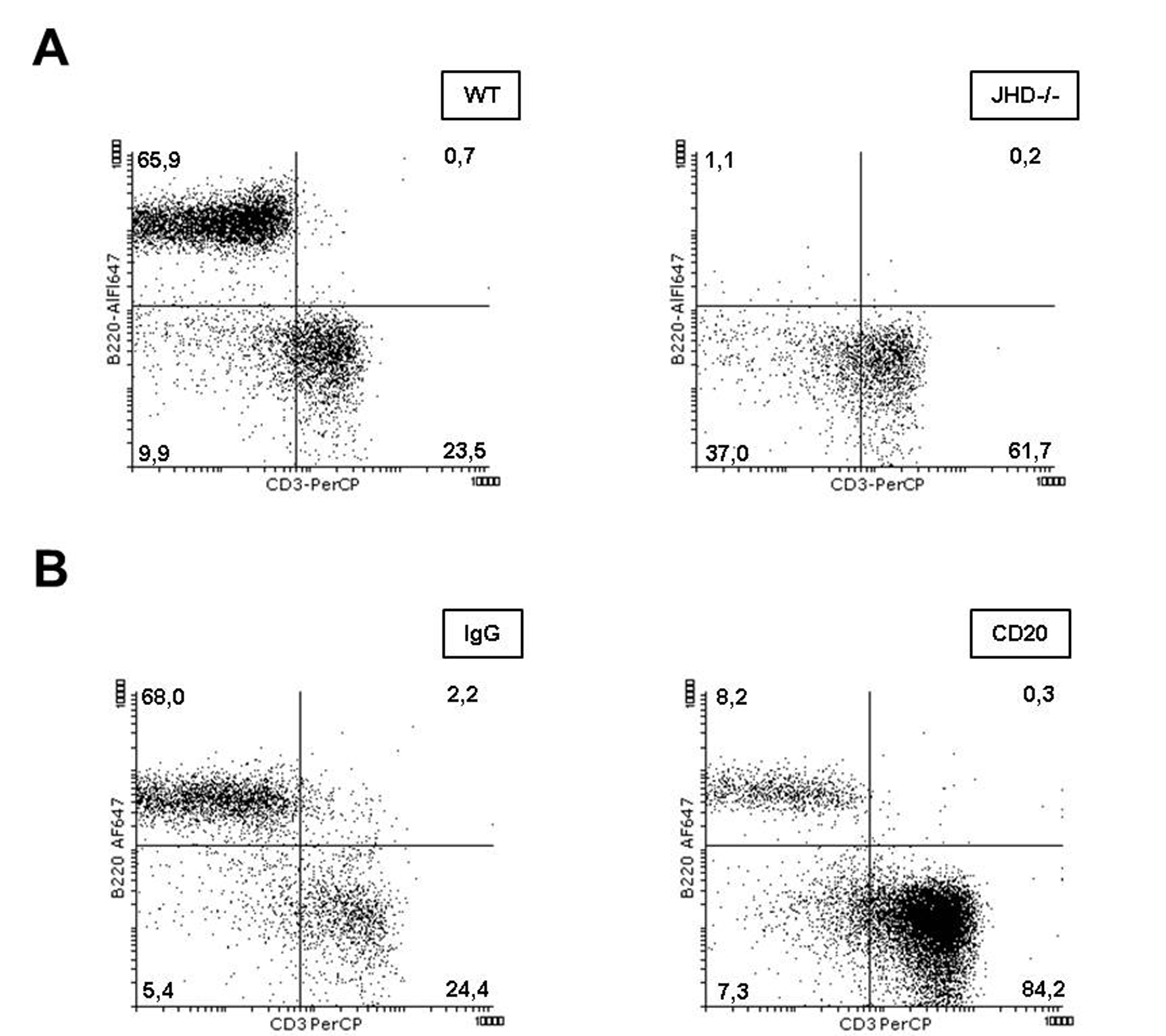
**

**Additional file 1: Figure S1.** *JHD^-/-^* mice and mice treated with anti-CD20 antibody display reduced B cell numbers when compared with corresponding control mice. **A,** Visualization (dot plot) of CD3^+^ T cells and B220^+^ B cells from peripheral blood of *JHD^+/+^* (**left**) and *JHD^-/-^* (**right**) mice as assessed by flow cytometry. **B,** Visualization (dot plot) of CD3^+^ T cells and B220^+^ B cells from peripheral blood of mice 24 hours after treatment with anti-IgG (**left**) or anti-CD20 (**right**) antibody as assessed by flow cytometry.

**
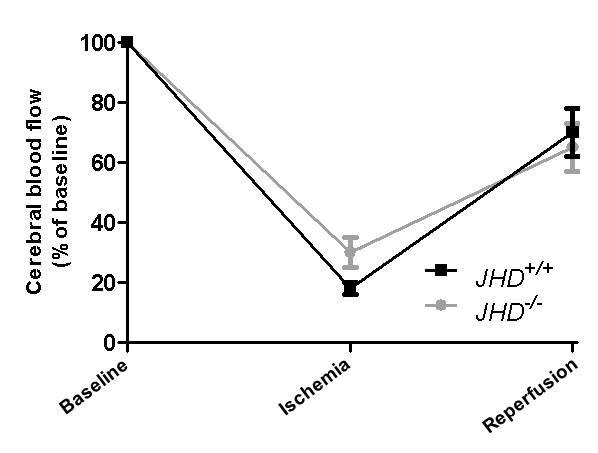
**

**Additional file 2: Figure S2.** Regional cerebral blood flow is comparable in *JHD^+/+^* and *JHD^-/-^* mice. Determination of rCBF using Laser Doppler flowmetry before (Baseline), after the occlusion of the middle cerebral artery (Ischemia) and again after the removal of the occluding filament (Reperfusion) in *JHD^+/+^* and *JHD^-/-^* mice. No significant differences in

rCBF were observed between the groups. n = 4.

**Additional file 1: Table S1. Dropout Rates of Mice Within the Distinct Genetic and Treatment Groups**

| Dropout rates | Day 1 post tMCAO | Day 3 post tMCAO |
| --- | --- | --- |
| WT + anti-IgG | 0/8 | n.a. |
| WT + anti-CD20 | 2/10 | n.a. |
| *JHD^+/+^* | 0/10 | 0/9 |
| *JHD^-/-^* | 4/13 | 0/11 |
| *Rag1^-/-^* | 1/11 | n.a. |
| *Rag1^-/-^* AT T cells | 0/8 | n.a. |
| *Rag1^-/-^* AT B cells | 2/11 | n.a. |

AT, adoptive transfer; n.a., not applicable; tMCAO, transient middle cerebral artery occlusion; WT, wild-type.

**Supplemental Methods**

**Flow cytometry**

For flow cytometry analysis of peripheral immune cells, 50 μL blood was used. Red blood cells were lysed using RBC lysis buffer (BioLegend) following the manufacturer’s instructions. The percentage of CD3^+^ (anti-CD3-PerCP, BioLegend) and B220^+^ (anti-B220-AF647, BioLegend) cells was quantified using a FACSCalibur (Becton Dickinson) flow cytometer.

**Laser-Doppler Flowmetry**

Laser-Doppler flowmetry (Moore Instruments) was performed in *JHD^+/+^* and *JHD^-/-^* mice before (Baseline), during (Ischemia) and immediately after tMCAO (Reperfusion). Regional cerebral blood flow was measured in the area of the right MCA (6 mm lateral and 2 mm posterior from bregma).
